# Loneliness and mental health during the COVID-19 pandemic in the Dutch general population: The moderating role of psychological flexibility

**DOI:** 10.1016/j.heliyon.2024.e37172

**Published:** 2024-08-30

**Authors:** Jasper Tilburg, Marianne Simons, Tim Batink, Mayke Janssens, Sanne Peeters, Johan Lataster, Nele Jacobs, Jennifer Reijnders

**Affiliations:** a Faculty of Psychology, Open Universiteit, Heerlen, the Netherlands; b Department of Psychiatry and Psychology, School for Mental Health and Neuroscience, Maastricht University Medical Centre, Maastricht, the Netherlands; c GGz centraal, Amersfoort, the Netherlands

**Keywords:** Loneliness, Mental health, Well-being, Psychological flexibility, COVID-19, Social isolation

## Abstract

Previous studies conducted during the COVID-19 pandemic found an increased risk of loneliness due to measurements taken by governments to limit social interaction. The current study addresses the relationship between loneliness and both positive and negative mental health in times of the COVID-19 pandemic and the role of psychological flexibility.

Data was collected in the Dutch general population (18+) with two online questionnaires (T1-T2), with an interval of four weeks. A sample of 340 participants varying in age from 18 to 83 years (M_age_ (SD): 52.83 (13) and 61.8 % female), filled in both questionnaires. Loneliness and (the six core processes of) psychological flexibility were measured at T1 and negative mental health (depression, anxiety and stress) and positive mental health (emotional, psychological and social well-being) at T2.

Regression analyses indicated a positive (prospective) relation between loneliness and depression, anxiety and stress and a negative (prospective) relation between loneliness and emotional, psychological and social well-being. Psychological flexibility had a moderating effect on the relation between loneliness and depression but not on the relation between loneliness and respectively anxiety, stress or well-being. Looking at the six core processes of psychological flexibility, being present and committed action were significant moderators of the relation between loneliness and depression.

We conclude that while it has yet to be determined whether the moderating relationships found in this study hold beyond COVID-19 conditions, the findings do provide support for the benefits of psychological flexibility to intervene in situations where external circumstances or psychological challenges are beyond one's control.

## 1 Introduction

The COVID-19 pandemic has changed our world perspective within a short time period. In addition to the physical health risks of the virus, the enforced measures have had a considerable impact on mental health [[1], [2], [3]]. As many societies took measures to keep people physically separated, concerns have been raised regarding the impact of these measures on well-being [4] and more particularly on the prevalence of loneliness [5,6]. After all, loneliness has been consistently linked to negative mental health variables [[7], [8], [9]]. The current study aims to generate more knowledge about coping with these measures taken by governments during the COVID-19 and maybe future pandemics, that limit social interaction. The main focus of the study concerns the role of *psychological flexibility* - the ability to deal flexibly and workably with the problems one encounters, while focusing on the things that matter [[10], [11], [12]] - and whether this equips people to better anticipate on the effects of measures that cannot be avoided and are likely to induce feelings of loneliness.

### 1.1 Loneliness and mental health

Loneliness is characterized by loss and disappointment that arises from a perceived gap between a person's desired and actual social network [13,14]. Research shows that a reduction in social activity increases the risk of loneliness [[15], [16], [17], [18]]. These results are reflected in several studies conducted during the COVID-19 pandemic, finding increased risk of loneliness among both young adults [5,19] and older age groups [[20], [21], [22], [23], [24], [25]] and also research showing higher levels of loneliness during times of increased COVID-19 restrictions (national lock-down) in the UK [26].

Loneliness has been linked to negative mental health effects such as depressive symptoms [7,8,[27], [28], [29]], increased levels of stress, anxiety, and negative feelings [7,30] and reduced self-regulation and emotion regulation [9,31]. Furthermore, loneliness has been found to be associated with a lower quality of life, both physical and mental [32] and lowered life expectancy [8].

These findings address either positive aspects (e.g. quality of life) or negative aspects (e.g. depression) of mental health. The dual continuum model of mental health [33,34] conceptualizes these two aspects of mental health as partly independent dimensions or 'continua', i.e., a continuum of mental well-being, ranging from languishing (low mental well-being) to flourishing (high mental well-being), and a continuum of psychopathology, ranging from the presence or absence of symptoms of psychopathology. The current study includes both dimensions and aims to explore how psychological flexibility can affect either the relation between loneliness and negative mental health outcomes (depression, anxiety and stress) or the relation between loneliness and positive mental health (well-being). We also include two measurement points to provide some evidence for prospective relations between loneliness and negative and positive mental health.

Following aforementioned research findings, we assume a positive prospective relation between perceived loneliness and negative mental health outcomes (depression, anxiety and stress) (hypothesis 1). Although the positive dimension of mental health has been less often the focus of research than negative mental health outcomes, previous research found negative associations between loneliness and well-being [25,[35], [36], [37]]. In the literature three separate sub-dimensions of well-being are distinguished [38,39]. *Emotional* well-being refers to life satisfaction and positive feelings such as happiness, interest, and pleasure in life [40,41]. *Social* well-being concerns someone's functioning within one's community and in society in general [40,42]. The third sub-dimension *psychological* well-being is usually described as effective and optimal functioning and includes aspects such as autonomy and self-actualisation [40,43]. Limited studies have addressed associations between these separate sub-dimensions and loneliness. In student populations, a negative association between loneliness and psychological well-being was found [44,45]. Stieger et al. (2021) found a negative association between loneliness and emotional well-being in an adult population during COVID-19 [46]. In older populations loneliness was found negatively associated with all three subdimensions [47,48]. We assume that, in line with these studies, a negative prospective relation between loneliness and each of the three subdimensions of well-being exists (hypothesis 2).

### 1.2 Psychological flexibility

Feelings of loneliness can motivate individuals to strengthen their existing social relationships or build new ones, in order to diminish these negative feelings of loneliness [8]. However, the enforced social restrictions during the COVID-19 pandemic calls for a different strategy. Such a strategy may be found in *psychological flexibility*, the core concept of Acceptance and Commitment Therapy (ACT, [12]). The emphasis of psychological flexibility lies on accepting personal experiences, rather than trying to change the content or intensity of these experiences [49]. Psychological flexibility arises from six core processes: acceptance (being open to inner experiences/feelings), defusion (observing thoughts without seeing them as the truth), being present (mindful awareness), self as context (flexible self-awareness and perspective taking), values (connection to personal values), and committed action (action based on personal values) [49].

In support of the above, it appears that people with greater psychological flexibility are better equipped to change emotional and physiological responses to align with the requirements of changing environmental conditions [50]. Also, they are better in shifting mindsets or behavioral patterns when these approaches undermine personal or social functioning [51] and are better able to balance life demands [52]. In addition, meta-analyses show that ACT-interventions, aimed at increasing psychological flexibility, are effective for improving mental health outcomes among diverse clinical and non-clinical populations [[53], [54], [55], [56], [57], [58], [59]]. Recent studies during COVID-19 showed that psychological flexibility is positively related with well-being and negatively with depression, anxiety, and distress [60]. Also, Pakenham and colleagues showed that psychological flexibility mitigated the negative impacts of COVID-19 risk factors (e.g. lockdown duration, family infected, increase in domestic violence and in unhealthy lifestyle behaviours) on negative mental health aspects [61]. Smith and colleagues found similar results showing that psychological flexibility can protect against the adverse effects of social isolation on anxiety, depression, distress. When considering positive outcome measures, psychological flexibility can enhance the positive impact of reduced social isolation on well-being. By increased social isolation, psychological flexibility did no longer have this beneficial effect [62].

Considering the previous findings showing that psychological flexibility can act as a buffer on negative mental health aspects, we expect that psychological flexibility plays a moderating role in the prospective positive relation between loneliness and depression, anxiety and stress (hypotheses 3) by mitigating this relation. For positive mental health we assume that psychological flexibility plays a moderating role as well, by weakening the prospective negative relation between loneliness and well-being (hypotheses 4), as there is some evidence that psychological flexibility can have positive effects.

In addition to the investigation of psychological flexibility as a moderator of the prospective relation between loneliness and respectively negative mental health outcomes (depression, anxiety and stress) and positive mental health outcome (well-being), the six core processes (acceptance, defusion, being present, self as context, values and committed action) will be investigated as possible moderators by means of explorative analyses. It could be argued that the COVID-19 pandemic can be considered a situation in which the external conditions leading to loneliness and psychological distress are beyond one's control and cannot be altered. This limits the possible impact of committed action based on one's own values. It is therefore assumed that the core processes representing the acceptance part in ACT (acceptance, defusion, being present, and self as context) will have stronger moderating effects.

### 1.3 Relevance

The current study aims to add to the understanding of the relationship between loneliness and both the positive and negative aspects of mental health outcomes in times of the COVID-19 pandemic, and contribute to the development of effective psychological strategies and interventions, as pandemics and freedom-restricting measures could remain part of human life. However, as other circumstances that cannot be easily altered – e.g. chronical disease or certain other life events – can also induce feelings of loneliness as well, the relevance of this study extends beyond particular circumstances of a pandemic.

## 2 Method

### 2.1 Design and sample

Data was collected in the Dutch population during the COVID-19 pandemic by graduate students of the Open Universiteit in the Netherlands. This study was part of a larger research line collecting data about psychological flexibility and well-being in the general Dutch population. An online survey was used by which quantitative data was collected at two test moments with four weeks in between. Inclusion criteria were age of 18 and older, residing in the Netherlands and sufficient knowledge of the Dutch language. In this study data was used from participants who completed both measurement 1 (T1) and 2 (T2). Demographics of the study sample are described in the Results section.

### 2.2 Procedure

Graduate students recruited participants via the university's website, social media, and their personal networks. People interested in participating, received an email with an information letter explaining the aims and procedure of this study and a personal link to the online questionnaire and consent form [63]. After completion of the first questionnaire (T1), participants received an invitation to complete the second questionnaire 4 weeks later (T2). Participants could withdraw from the study at any time without reason or consequences. Personal data (email address) was deleted after completing both measurements and the data was analyzed anonymously by means of tokens. The study content and procedures were approved by the ethics committee of the Open Universiteit (U2018/09690/MQF) and followed the American Psychological Association Ethical principles of psychologists and code of conduct [64].

### 2.3 Measures

#### 2.3.1 Demographic variables and confounders

The questionnaire started with several items measuring demographical characteristics and relevant confounders. In addition to gender and age, education level (0 = low: up till secondary school or vocational education, 1 = high: at least an undergraduate degree) and relationship status (single: 0 = no, 1 = yes) were included in this study as confounders, as previous studies show associations between these demographic variables and loneliness [13,17,[65], [66], [67]].

#### 2.3.2 Loneliness

Loneliness was measured with the Loneliness Questionnaire (LQ, [68]), consisting of 11 items, five positively and six negatively stated. Responses to the items such as ‘I often feel abandoned’ and ‘I have a lot of people around me who I can trust completely’, were rated on a 5-point scale ranging from ‘Yes, I totally agree’ (1) to ‘No, I totally disagree’ (5). Neutral and negative answers on positively stated items are scored as ‘1’ and positive answers as ‘0’. Consequently, neutral and positive answers on negatively stated items are scored as ‘1’ and negative answers as ‘0’. Adding up theses scores of the 11 items yields a total scale score ranging from 0 to 11. The higher the score, the higher the perceived loneliness. A score of three or higher was indicative for moderate loneliness; a score of nine or higher for severe loneliness [68].

#### 2.3.3 Depression, anxiety and stress

The shortened version of the Depression Anxiety Stress Scale (DASS-21, [69]), consisting of three subscales: depression (items 3, 5, 10, 13, 16, 17, 21) anxiety (items 2, 4, 7, 9, 15, 19, 20) and stress (items 1, 6, 8, 11, 12, 14, 18) was used to measure negative aspects of mental health. Responses to the items such as ‘I was unable to get excited about anything’ (depression), ‘I felt like I was about to panic’ (anxiety), and ‘I tended to overreact to situations’ (stress), were rated on a Likert scale ranging from ‘not at all or never applicable’ (0) to ‘very sure or mostly applicable’ (3). The subscale scores for depression, anxiety and stress are used in this study. They are calculated by adding the scores of the corresponding items per subscale with higher scores indicating higher depression, anxiety or stress levels.

#### 2.3.4 Well-being

Well-being was measured with the Dutch Continuum Mental Health Short Form (MHC-SF; [39]), measuring emotional well-being (items 1 to 3), social well-being (items 4 to 8) and psychological well-being (items 9 to 14). Responses to the items such as ‘In the past month, how often did you feel satisfied?’ (emotional well-being), ‘In the past month, how often did you feel that you were part of a community?’ (social well-being) and ‘In the past month, how often did you feel that you liked most aspects of your personality?’ (psychological well-being) were scored on a 6-point Likert scale from ‘never’ (0) to ‘every day’ (5). Mean scores for each subscale was calculated with a higher score indicating higher emotional, social or psychological well-being.

#### 2.3.5 Psychological flexibility

Psychological flexibility was measured with the Flexibility Index Test (FIT-60; [70,71]) consisting of 60 items, evenly distributed over the six subscales: acceptance, defusion, being present, self as context, values, and committed action. Responses to the items such as ‘My thoughts cause me discomfort or emotional pain’ (acceptance), and ‘I observe my feelings without getting lost in them’ (defusion) were scored on a 7-point Likert scale ranging from (0) ‘totally disagree’ to (6) ‘totally agree.’ The negatively formulated items were reversed coded. The total score ranges from 0 to 360 and the subscale scores from 0 to 60 with a higher score indicating a higher level of (sub-dimension of) psychological flexibility.

### 2.4 Data analysis

Statistical analyses were performed with SPSS (V27, IBM Corp. NY). A comparison was made for the demographical characteristics and core variables between drop-outs (participants who only filled in the questionnaire at T1) and completers (participants who filled in the questionnaire both at T1 and T2) and Pearson's χ2 and an independent T-tests were performed to detect possible attrition bias. Reliability of scales (Cronbach's alpha and McDonald's Omega) was examined and (Pearson's) correlations between main study variables were computed. The variables loneliness, depression, anxiety, stress, psychological well-being, social well-being, emotional well-being, psychological flexibility and the six sub-dimensions were standardized by means of z-scores to ensure comparison of the different measurement scales used. To check for outliers a boxplot was drawn up, and to check for multicollinearity a VIF-test was performed.

Multiple hierarchical regression analyses (enter, stepwise) were performed to test hypotheses 1 and 2, with loneliness as predictor (measured at T1) and respectively depression, anxiety and stress (measured at T2, model 1a,b,c) and the sub-dimensions of well-being (measured at T2, model 2a,b,c) as dependent variables. To test hypotheses 3 and 4, interaction variables loneliness (T1)*psychological flexibility (T1) were added to these two regression models. If the moderation analyses proved to be significant, we additionally investigated, as explorative analyses, the six individual core processes of psychological flexibility in a similar way in separate regression analyses. In all analyses the confounders age, gender, educational level and relationship status were included. In addition, the outcome variable at T1 was included to control for baseline measurement.

A significance level of 5 % was used. To interpret the significant interaction effects, the effects were plotted using tests in accordance with Dawson (2014) [72].

## 3 Results

### 3.1 Descriptives

Table 1 displays demographics and core variables of participants that filled out both questionnaires (completers, n = 340) and of those that only filled out the first questionnaire (drop-outs, n = 99). Significant differences between these two samples were found for age (drop-outs: *Mean* = 49.51; completers: *Mean* = 52.83) and level of education (drop-outs: 44.4 % low; completers: 23.8 % low) but not on the core variables in this study. The completers sample of 340 participants was used for further analyses. Of these 340 participants, 61.8 % was female (n = 210), varying in age from 18 to 83 years (*Mean(SD)* = 52.83 (13.00)). The educational level varied from lower vocational education to a graduate degree, with 259 (76.2 %) participants having a higher educational level. The vast majority (81.8 %) of the participants were in a relationship.

**Table 1:** Demographic and core variables completers and drop-outs.

Demographic variables	only T1 (drop-outs)		T1 and T2 (completers)		
	n = 99		n = 340		
	*n*	%	*n*	%	χ^2^/*t*
Gender					*χ*^*2*^ (1) = .02, *p* = .88
Female	62	62.6	210	61.8	
Male	37	37.4	130	38.2	
Other	0	–	–	–	
Single					*χ*^*2*^ (1) = .02, *p* = .89
no	80	82.5	275	81.8	
yes	17	17.5	61	18.2	
Educational level					*χ*^*2*^ (1) = 16.01, *p* < .01**
Low	44	44.4	81	23.8	
High	55	55.6	259	76.2	
Age (Mean, SD)	49.51	13.73	52.83	13.00	*t* (437) = 2.21, *p*=.03*
Core variables					
	*M*	SD	*M*	SD	
Loneliness	2.74	3.22	2.80	3.23	*t* (437) = -.18, *p* = .86
Depression	2.55	3.35	2.53	3.69	*t* (437) = -.03, *p* = .98
Anxiety	1.85	2.65	1.92	3.03	*t* (437) *=* -.22, *p =* .82
Stress	3.90	3.93	4.11	4.03	*t* (437) *= -*.47, *p =* .64
Emotional well-being	3.91	.86	3.72	.95	*t* (437) = 1.71, *p* = .09
Psychological well-being	3.62	.95	3.44	.95	*t* (437) = 1.68, *p* = .09
Social well-being	2.64	1.01	2.68	.99	*t* (437) = -.28, *p* = .78
Psychological flexibility	246.83	45.92	247.12	44.32	*t* (437) = -.06, *p* = .95

*p < .05, **p < .01.

The descriptive statistics and correlations of the core variables are presented in Table 2. As expected, significant negative correlations were found between loneliness (T1) and emotional well-being (r = −.466), psychological well-being (r = −.444) and social well-being (r = −.405) (T2) and significant positive correlations between loneliness (T1) and depression (r = .594), anxiety (r = .394) and stress (r = .430) (T2). For psychological flexibility (T1) significant positive correlations were found with emotional well-being (r = .515), psychological well-being (r = .600) and social well-being (r = .403) (T2) and significant negative correlations with depression (r = −.657), anxiety (r = −.511) and stress (r = −.582) (T2). There is a significant negative correlation (r = −.522) between the predictor loneliness (T1) and the moderator psychological flexibility (T1).

**Table 2:** Descriptive statistics and correlations of the sample (n = 340) included in our main analyses.

	α	ω	Mean	SD	Min	Max	Correlations													
							1	2	3	4	5	6	7	8	9	10	11	12	13	14
1.Loneliness (T1)	.88	.88	2.79	3.22	0	11	–	−.522	−.448	−.423	−.333	−.350	−.662	−.350	.594	.394	.430	−.466	−.444	−.405
2. PF (T1)	.94	1.00	247.06	44.63	76	344		–	.874	.863	.764	.842	.720	.724	−.657	−.511	−.582	.515	.600	.403
3.Acceptance (T1)	.82	.82	40.51	9.91	0	60			–	.774	.660	.682	.530	.482	−.569	−.503	−.547	.431	.478	.287
4. Defusion (T1)	.90	.91	37.61	12.77	1	60				–	.636	.680	.463	.431	−.531	−.468	−.541	.404	.402	.273
5. Self as Context (T1)	.50	–	35.23	7.40	11	55					–	.560	.443	.421	−.490	−.364	−.469	.334	.399	.214
6. Being Present (T1)	.80	.78	42.51	9.37	8	60						–	.510	.578	−.478	−.376	−.447	.364	.483	.308
7. Values (T1)	.76	.76	47.27	7.06	24	60							–	.673	−.629	−.424	−.464	.542	.578	.463
8. Committed action (T1)	.83	.83	43.93	8.74	7	60								–	−.511	−.310	−.323	.454	.624	.459
9. Depression (T2)	.89	.89	2.40	3.50	0	21									–	.656	.655	−.655	−.550	−.377
10. Anxiety (T2)	.83	.83	1.62	2.62	0	15										–	.747	−.426	−.310	−.189
11. Stress (T2)	.89	.89	3.74	3.76	0	19											–	−.489	−.397	−.243
12.Emo well-being (T2)	.83	.83	3.70	.94	.00	5												–	.682	.576
13. Psy well-being (T2)	.86	.86	3.58	.93	.67	5													–	.704
14. Soc well-being (T2)	.78	.78	2.82	1.01	.40	5														–

PF=Psychological flexibility, Emo = Emotional, Psy = Psychological, Soc = Social. **All correlations *p < .01***.

Cronbach's alpha and McDonald's Omega values for the used scales were all >.70 and can therefore be considered sufficiently reliable [73,74]. Except for subscale self as context from the FIT-60 with Cronbach's alpha of .50 and McDonald's Omega could not be estimated due to negative or zero term covariances. As we use this subscale only for the explorative analyses and the total score of the FIT-60 (with alpha and omega >90) for the main analyses we considered the total scale sufficiently reliable for the purpose of our study.

### 3.2 Results regression analyses

Regression analysis found a positive (prospective) association between loneliness (T1) and depression (T2) (*β* = .220, *p* < .001), between loneliness (T1) and anxiety (T2) (*β* = .143, *p* < .001) and between loneliness (T1) and stress (T2) (*β* = .170, *p* < .001) which supports hypothesis 1. In model 1a (depression as dependent variable) the covariates relationship status (*β* = .069, *p* = .041), and depression at T1 (*β* = .648, *p* < .001) were significant. In model 1b (anxiety as dependent variable) anxiety at T1 was significant (*β* = .647, *p* < .001). In model 1c (stress as dependent variable) stress at T1 was significant (*β* = .656, *p* < .001).

Negative (prospective) associations were found between loneliness (T1) and respectively emotional well-being (*β* = −.189, *p* < .001), psychological well-being (*β* = −.148, *p* < .001) and social well-being (*β* = −.120, *p* = .002), which supports hypotheses 2. In model 2a,b,c (respectively emotional, psychological and social well-being as dependent variable) also the outcome variables at T1 were significant (*β* = .634, *p* < .001; *β* = .696, *p* < .001; *β* = .729, *p* < .001).

**Fig. 1:**
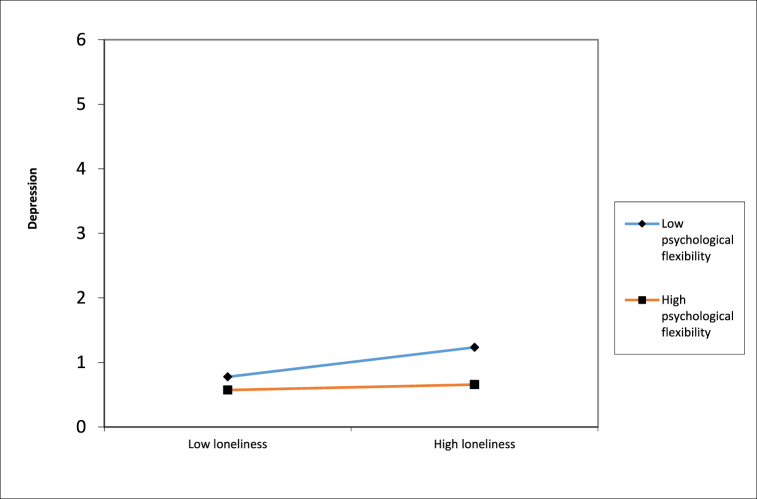
Two-way interaction between loneliness and depression moderated by psychological flexibility.

With regard to hypothesis 3 (moderation of psychological flexibility) Table 3 shows that the model for depression was significant, showing a significant positive (prospective) association between loneliness and depression (*β* = .136, *p* < .001), moderated by psychological flexibility (*β* = −.111, *p* = .002). This indicates that the association between loneliness and depression is stronger for people with low psychological flexibility, supporting hypothesis 3. In Fig. 1a a graphical representation of this moderation effect is shown.

**Table 3:** Hierarchical multiple regression analysis of the moderating effect of psychological flexibility between loneliness and depression, anxiety and stress.

dependent variable	Depression (DASS)(T2)			Anxiety (DASS)(T2)			Stress (DASS)(T2)		
predictor	*B*	SE(B)	p-value	*B*	SE(B)	p-value	*B*	SE(B)	p-value
**Step 3**									
Gender	.044	.064	.158	.012	.080	.758	−.006	.075	.878
Education	.003	.074	.918	.035	.092	.369	.057	.087	.119
Single	**.088**	.083	.007**	−.016	.104	.696	.005	.097	.896
Age	−.028	.002	.383	−.003	.003	.941	−.027	.003	.467
Depression (T1)	**.519**	.043	<.001**	–	–	–	–	–	–
Anxiety (T1)	–	–	**-**	**.577**	.045	<.001**	–	–	–
Stress (T1)	–	–	**-**	–	–	–	**.565**	.047	<.001**
Loneliness (T1)	**.136**	.040	<.001**	.066	.049	.179	**.100**	.046	.030**
PF (T1)	**−.195**	.043	<.001**	**−.159**	.051	.002**	**−.200**	.050	<.001**
Loneliness x PF	**−.111**	.030	.002**	−.046	.037	.296	.002	.035	.960
	Adj. *R*^*2*^ = .685 *F* (8,335) = 92.073 *p* < .001**				Adj. *R*^*2*^ = .517 *F* (8,335) = 45.838 *p* < .001**		Adj. *R*^*2*^ = .573 *F* (8,335) = 57.284 *p* < .001**		

PF= Psychological Flexibility. Model: Enter: step 1: covariates, step2: loneliness and PF added, step 3: Loneliness*PF added. **p < .01. Gender (0 = male, 1 = female); Single (0 = no, 1 = yes); education (0 = low; 1 = high). Standardized scores were included for depression, anxiety, stress, psychological flexibility, and loneliness.

Table 4 shows that the models for the sub-dimensions of mental well-being were significant. There were significant negative associations between loneliness and emotional well-being (*β* = −.137, *p* < .001), psychological well-being (*β* = −.093, *p* = .038), and social well-being (*β* = −.125, *p* = 005), and significant positive associations between psychological flexibility and psychological well-being (*β* = −.146, *p* = .004). In contrast to depression, the moderator loneliness*psychological flexibility was not significant for emotional well-being (*β* = .051, *p* = .221), psychological well-being (*β* = .026, *p* = .517), and social well-being (*β* = −.042, *p* = .283).

**Table 4:** Hierarchical multiple regression analysis of the moderating effect of psychological flexibility between loneliness and the sub-dimensions of well-being.

dependent variable	Emotional well-being (T2)			Psychological well-being (T2)			Social well-being (T2)		
predictor	*B*	SE(B)	p-value	*B*	SE(B)	p-value	*B*	SE(B)	p-value
**Step 3**									
Gender	−.002	.076	.958	.033	.073	.357	.007	.072	.843
Education	−.039	.088	.297	.053	.084	.142	.040	.083	.255
Single	−.070	.099	.068	.012	.094	.751	.018	.093	.625
Age	.007	.003	.858	−.008	.003	.819	−.057	.003	.106
Emo (T1)	**.599**	.044	**<.001**^**⁎⁎**^						
Psy (T1)				**.618**	.046	**<.001**^**⁎⁎**^			
Soc (T1)						.	**.719**	.040	**<.001**^**⁎⁎**^
Loneliness (T1)	**−.137**	.047	**.004**^**⁎⁎**^	**−.093**	.045	**.038**^**⁎**^	**−.125**	.045	**.005**^**⁎⁎**^
PF (T1)	.082	.050	.097	**.146**	.051	**.004**^**⁎⁎**^	.033	.044	.453
Loneliness x PF	.051	.035	.221	.026	.034	.517	−.042	.033	.283
	Adj. *R*^*2*^ = .555 *F* (8,335) = 53.144 *p* < .001^**⁎⁎**^				Adj. *R*^*2*^ = .594 *F* (8,335) = 62.266 *p* < .001^**⁎⁎**^		Adj. *R*^*2*^ = .603 *F* (8,335) = 64.669 *p* < .001^**⁎⁎**^		

Emo = emotional well-being; Psy = psychological well-being; Soc = social well-being; PF=Psychological flexibility. Model: Enter: step 1: covariates, step2: loneliness and PF added, step 3: Loneliness*PF added. Gender (0 = male, 1 = female); Single (0 = no, 1 = yes); education (0 = low; 1 = high); Standardized scores were included for psychological flexibility, loneliness, emotional, psychological, and social well-being and depression, anxiety and stress.^∗^p < .05^∗∗^*p* < .01.

Finally, we further investigated the moderating effect of psychological flexibility on the relationship between loneliness and depression by looking at the six core processes separately. The results (see Table 5) show that the processes involving being present (β = −.126, p = .012) and commited action (β = −.091, p = .047) were significant moderators. Although the subscale commited action was marginal significant and caution is advised by interpreting this finding.

**Table 5:** Hierarchical multiple regression analysis of the moderating effect of the six ACT processes between loneliness and depression.

dependent variable	Depression (DASS)(T2)		
predictor	*B*	SE(B)	p-value
**Step 3**			
Gender	.061	.065	.055
Education	.016	.073	.616
Single	**.109**	.081	<.001**
Age	−.028	.002	.383
Depression (T1)	**.515**	.042	<.001**
Loneliness (T1)	.075	.048	.116
Acceptance (T1)	.000	.060	.994
Defusion (T1)	.048	.058	.395
Self as Context (T1)	**−.114**	.043	.008**
Being Present (T1)	−.004	.046	.938
Values (T1)	−.046	.054	.386
Action (T1)	**−.186**	.045	<.001**
Loneliness x Acceptance	−.103	.055	.122
Loneliness x Defusion	.007	.062	.912
Loneliness x Self as Context	−.028	.043	.563
Loneliness x Being Present	**.126**	.043	.012*
Loneliness x Values	−.080	.043	.125
Loneliness x Action	**−.091**	.043	.047*
	Adj. *R2* = .715 *F* (18,335) = 47.578, *p* < .001**		

Note. Model: Enter: step 1: covariates, step2: six ACT processes were added, step 3: moderators were added. *p < .05, **p < .01. Gender (0 = male, 1 = female); Single (0 = no, 1 = yes); education (0 = low; 1 = high). Standardized scores were included for depression, the subdimensions of psychological flexibility, loneliness, and the moderating variables.

## 4 Discussion

The aim of this study was to investigate the prospective relation between loneliness and respectively negative mental health (depression, anxiety and stress) and positive mental health (emotional, psychological and social well-being) and the role of psychological flexibility during COVID-19 in a Dutch general population sample. Hypothesis 1 (positive prospective relation between loneliness and depression, anxiety and stress) and hypothesis 2 (negative prospective relation between loneliness and emotional, psychological and social well-being) were supported by the results. Furthermore psychological flexibility had a moderating effect on the relation between loneliness and depression but not on the relation between loneliness and anxiety or loneliness and stress (hypotheses 3). Also there were no moderating effects on the relation between loneliness and emotional, psychological or social well-being (hypotheses 4). These results indicate that the (positive) relation between loneliness and depression is stronger for people with low psychological flexibility. As an exploratory analysis, we also looked at the six core processes separately. The results showed that the process of being present was a significant moderator of the relation between loneliness and depression and the process of committed action was marginally significant.

Findings from the current study were consistent with earlier studies that found a positive relation between loneliness and depression, anxiety, and stress [7,8,[27], [28], [29], [30]] and a negative relation between loneliness and the three sub-dimensions of well-being [25,[35], [36], [37]]. In our study both negative and positive aspects of mental health, in line with the dual continuum model of mental health, were included and also two measurements were used to give some indications for prospective relations.

The finding that psychological flexibility moderates the relation between loneliness and depression is consistent with the findings of Pakenham et al. (2020) [61] and Smith et al. (2020 [62]). Pakenham et al. (2020) found that psychological (in)flexibility moderated the relation between COVID-19 lockdown risk factors on stress, anxiety, and depression [61]. Smith et al. (2020) found that psychological inflexibility and intolerance of uncertainty moderated the effect of social isolation on negative mental health and also on well-being [62]. The study of Dawson & Golijani-Moghaddam (2020), although not performing moderator analyses, found that psychological flexibility provided additional explanatory power beyond the contribution of coping style variables in relation with well-being, depression, anxiety and COVID-19 distress and worry. Psychological flexibility can according to Dawson and colleague (2020) be understood as a higher-order response pattern that may facilitate the selection of coping strategies (and other behaviors) that are appropriate for the situation [60]. Our study confirms the findings of these previous studies for depression but not for anxiety and stress. A possible explanation for this is that in our study we used two measurement points and controlled for baseline outcome measures. Post-hoc analysis without controlling for outcome measures at T1 did show a significant moderating effect of psychological flexibility on the relation between loneliness and anxiety (β = −.128, p = .015) but not on the relation between loneliness and stress (β = −.065, p = .184). Another explanation might be the level of distress of the current study population is lower than previous studies and therefore showing slightly different results.

Interestingly and unexpectedly our findings show that psychological flexibility does not moderate the relation between loneliness and emotional, psychological and social well-being. This seems in contrast to the findings of Smith and colleagues (2020) [62]. Looking more closely at this study, psychological *in*flexibility was measured with the AAQ, which is measuring experiential avoidance which does not completely cover the concept of psychological flexibility described by the ACT model [75]. Another explanation might be that psychological flexibility and *in*flexibility do not represent opposite ends of a single dimension, but rather two separable dimensions of functioning worthwhile to be evaluated and modeled as distinct constructs [76]. This is in line with the study of Howell & Demuynck (2021), showing that both psychological flexibility and psychological inflexibility explain a significant part of variability in hedonic and eudemonic well-being [77]. This might explain the difference in findings regarding the moderating role of psychological flexibility on the relation between loneliness and well-being. Further research with different conceptualizations of psychological (in)flexibility can bring more clarity about this.

Another possible explanation for the difference may lie in the operationalization of the concept of positive mental health. In the earlier mentioned study by Smith et al. (2020) [62], well-being was operationalized using the WHO-5. The WHO-5, although initially developed as a measure of well-being, it is also applied as a assessment tool for depression with high sensitivity [78]. This may have resulted in an overlap between positive and negative mental health in their study. Another possible explanation may be found in the growing body of literature on the COVID-19 impact on social connectivity. Despite the mandatory physical distance during COVID-19 and feelings of loneliness that can arise from this, people can feel more emotionally and socially connected because they are 'all in this fight together' [[79], [80], [81]]. Many people have felt they are part of the community wide effort to slow the spread of the virus [79]. Such feelings may have helped mitigate the effect of loneliness on well-being. Perhaps the two-continuum model plays a role here; positive mental health and negative mental health are two independent but related constructs with each their own continuum and possibly their own mitigating factors. More research is needed.

Furthermore, the results of the explorative analyses showed that the core process of being present significantly moderated the prospective relation between loneliness and depression. The core process committed action was marginally significant and therefore caution by interpreting this findings is advised. Being present, or mindful awareness, is defined as a moment-to-moment non-judgmental acceptance of one's present experience [82]. The research findings may imply that mindful awareness is especially important in times of imposed measures beyond one's control, as in the COVID-19 pandemic. Mindfulness has been shown an important coping mechanism in other studies addressing the COVID-19 pandemic [[83], [84], [85]], but also in other circumstances in which things are beyond one's control, for example a chronic disease [86,87]. Pakenham and colleagues (2020) [61] investigated the different core processes of psychological flexibility and inflexibility and found, among others, moderating effects for committed action and lack of present moment although comparison is difficult due to different conceptualization of psychological (in)flexibility. The explorative analyses suggest that accepting the present experience, while also committed to valued action, increases resilience and flexibility during challenges beyond one's control. More research is needed.

Taken together, our study adds to the existing literature on the relation between loneliness and mental health with indications of prospective relations between loneliness and depression, anxiety, stress and well-being and also specifying well-being into three subcomponents. The findings provide further support for the importance of developing psychological flexibility during the COVID-19 pandemic or similar situations, specifically in relation to depression. This is consistent with the idea that people with greater psychological flexibility are better equipped to align with changing conditions and balance life demands [[50], [51], [52]] and in line with the large body of research emphasizing the crucial role of psychological flexibility in promoting psychological health and adaptation [51].

### 4.1 Strengths and limitations

The current study gives valuable information into the relation between loneliness, mental health and psychological flexibility. The main strength, in comparison to previous studies, is the design with two measurement points thus allowing to examine prospective relation between the constructs. Furthermore this study was conducted with validated questionnaires among a large population of adults between 18 and 90 years old. There are however a number of limitations that should be considered. A first concern is that, although our variables were measured over time, there were only two measurement points and loneliness was only measured once, so no definite conclusions can be drawn about directionality and the causal relationships between loneliness, psychological flexibility, and mental health outcomes. Longitudinal studies with more measurements over time are needed to give more insight into the causal relationships. Secondly, the demographic composition of the sample was not representative of the Dutch population. The majority of the participants were female, higher educated and in a relationship. Existing studies show that men, lower educated and single experience more loneliness [13,17,[65], [66], [67]]. Also the completers groups showed to be higher educated and of older age as the drop-outs group. This impacts the generalization of our results. Finally, we used self-reported measurements for all variables which are vulnerable to the effects of social desirability bias [88,89]. Besides this the FIT-60, which allowed us to make an exploratory distinction between the core processes of psychological flexibility, has not yet been validated as such and the subscale self-as-context needs further investigation because of low reliability probably due to multiple factor loadings within the subscale. The scale also contains a large number of items that make significant demands on the understanding and ability to reflect of the respondents. Other measures, e.g. AAQ, are more concise and may therefore be more accessible for respondents.

### 4.2 Future research and implications

The current study includes several directions for further research. Monitoring respondents over a longer period of time will shed light on the direction of found associations between loneliness, mental health and psychological flexibility. The results also prompt further research on how to improve people's positive mental health in times of social isolation One possibility is to further investigate which factors, in addition to psychological flexibility, may have a mitigating effect on the negative effects of loneliness on well-being and to include both psychological flexibility and psychological inflexibility as two separate variables [76]. Future research may also focus on the unique contribution and role of the six core processes of psychological flexibility, which may have different relevance in various situations and reliable measurements of these core processes.

Psychological flexibility equips people to manage uncontrollable situations such as the COVID-19 pandemic. Our study was conducted during a very unique period but the results can be extended to other circumstances in which people experience restrictions, whether imposed from the outside world or arising from personal life events and conditions such as chronic or life-threatening diseases [90,91]. In terms of the development of effective psychological strategies or interventions, a widespread and easy assessable ACT intervention, which focuses on being present, feeling the distress and commit to values action, rather than changing the content or intensity of the distress, offers the opportunity to intervene in situations where external circumstances or psychological challenges are beyond one's control.

### 4.3 Conclusion

In summary, the current study found positive prospective relations between loneliness and depression, anxiety and stress and negative prospective relations between loneliness and emotional, psychological and social well-being. Psychological flexibility had a moderating effect on the relation between loneliness and depression. This is consistent with the concept of psychological flexibility within ACT, which is not about symptom reduction but about developing the flexibility to cope with challenging life situations even when the circumstances or psychological challenges are beyond one's control. While it has yet to be determined whether the moderating relationships found in this study hold beyond COVID-19 conditions, the findings do provide support for the benefits of psychological flexibility in changing the relationship between loneliness and depression. It gives us insight into a potentially effective psychological intervention for situations in which loneliness is becoming more prevalent regardless of the underlying causes.

## Ethical approval

The study was reviewed and approved by the ethics committee of the Open University (U2018/09690/MQF). All participants provided informed consent to participate in the study.

## Statement of conflict of interest

The authors declare that they have no relevant financial or non-financial interests that relate to the research described in this paper. Author TB declares that providing ACT courses and trainings from his company has no conflict with the research described in this paper.

## Statement of funding

No funding was received for conducting this study.

## Data availability statement

All data from this study is available on request by the researchers.

## CRediT authorship contribution statement

**Jasper Tilburg:** Writing – original draft, Formal analysis, Conceptualization. **Marianne Simons:** Writing – review & editing, Data curation, Conceptualization. **Tim Batink:** Writing – review & editing. **Mayke Janssens:** Writing – review & editing. **Sanne Peeters:** Writing – review & editing. **Johan Lataster:** Writing – review & editing, Supervision. **Nele Jacobs:** Writing – review & editing, Supervision. **Jennifer Reijnders:** Writing – original draft, Formal analysis, Data curation, Conceptualization.

## Declaration of competing interest

The authors declare the following financial interests/personal relationships which may be considered as potential competing interests:Tim Batink reports a relationship with ACT in actie that includes: equity or stocks. If there are other authors, they declare that they have no known competing financial interests or personal relationships that could have appeared to influence the work reported in this paper.
